# Supplementary material for: Radical pair model for magnetic field effects on NMDA receptor activity
Source: Sci Rep. 2024 Feb 13;14:3628. doi: 10.1038/s41598-024-54343-y (PMC10864372; doi:10.1038/s41598-024-54343-y)
Supplement: Supplementary file 1 — Supplementary Information. [file 41598_2024_54343_MOESM1_ESM.pdf]

Radical Pair Model for Magnetic Field Effects on NMDA Receptor Activity
Supplementary Information

Parvathy S Nair[1,✉], **Hadi Zadeh-Haghighi**[2,3,4,✉], **and Christoph Simon**[2,3,4,✉]

[1]Department of Physics, Indian Institute of Science Education and Research (IISER) Tirupati, Tirupati-517507, Andhra Pradesh, India
[2]Department of Physics and Astronomy, University of Calgary, Calgary, AB T2N 1N4, Canada.
[3]Institute for Quantum Science and Technology, University of Calgary, Calgary, AB T2N 1N4, Canada.
[4]Hotchkiss Brain Institute, University of Calgary, Calgary, AB T2N 1N4, Canada.
✉email: parvathysnair@students.iisertirupati.ac.in; hadi.zadehhaghighi@ucalgary.ca; csimo@ucalgary.ca

A. Magnesium Isotope Effect

We performed calculations of FSY for the RPs with the spinful and spinless isotopes taken separately to understand the potential effects of the spin on the behaviour of the system.

Ser Pathway

Fig.1 shows the quantity S for the radical pair with the serine oxyradical plotted against the reaction rate, k, and relaxation rate, r. Both calculations consider the contribution from the nucleus of Serine oxyradical that has the highest HFCC, . Fig.1a shows the behaviour when the calculations consider the spinless isotopes, ^{24}Mg and ^{26}Mg. Fig.1b shows the case where the HFI contribution from the spinful isotope, ^{25}Mg, is considered. We find that higher values of S are found when only the spinful isotope is considered for the calculation.

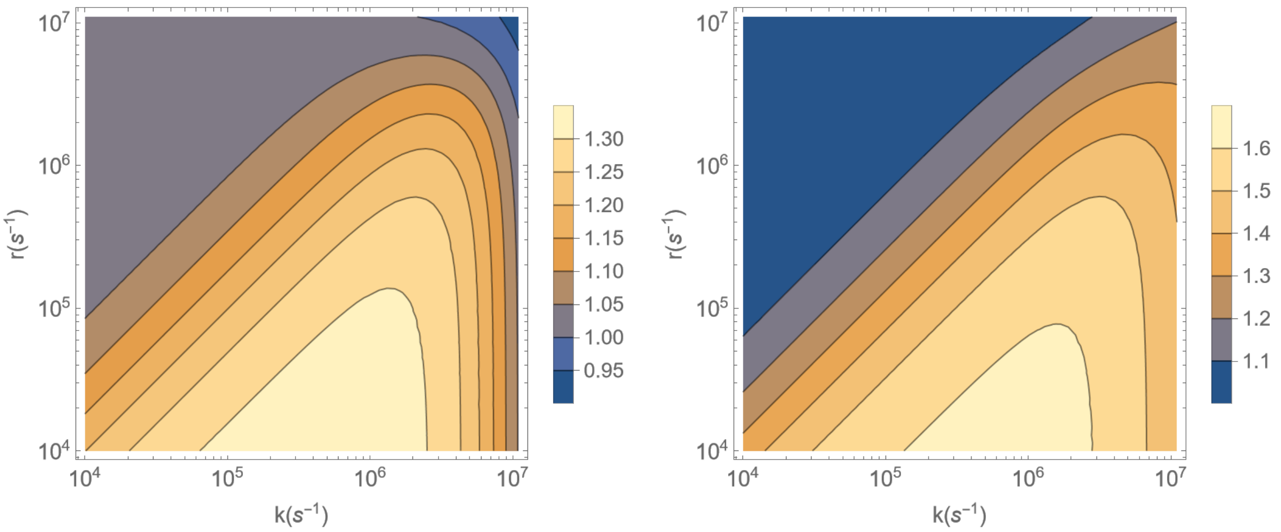

(a) (b)

Figure 1. Ratio of the fractional singlet yield percentages, S plotted against $k(\mathrm{s}^{-1})$ and $r(\mathrm{s}^{-1})$ for Ser radical case: (a) System containing only ^{24}Mg and ^{26}Mg isotopes is considered; (b) System containing ^{25}Mg isotope with the HFCC, $a_B = -11.22$ mT has been considered.

An example of the variation of fractional singlet yield percentage with magnetic field strength from 0.15 mT to 100 mT for a choice of k and r from the feasible region is given in Figs. 2a and 2b. We choose $k = 2 \times 10^6$ s^{-1} and $r = 3 \times 10^5$ s^{-1} for the

same. We find that the behaviour of the system is significantly different between the spinless and spinful cases. The singlet yield at low field are substantially lower for the spinful case. There are sudden spikes in the value at certain MF strengths, as well, which can be associated with ethe igenenergies of certain states crossing over.

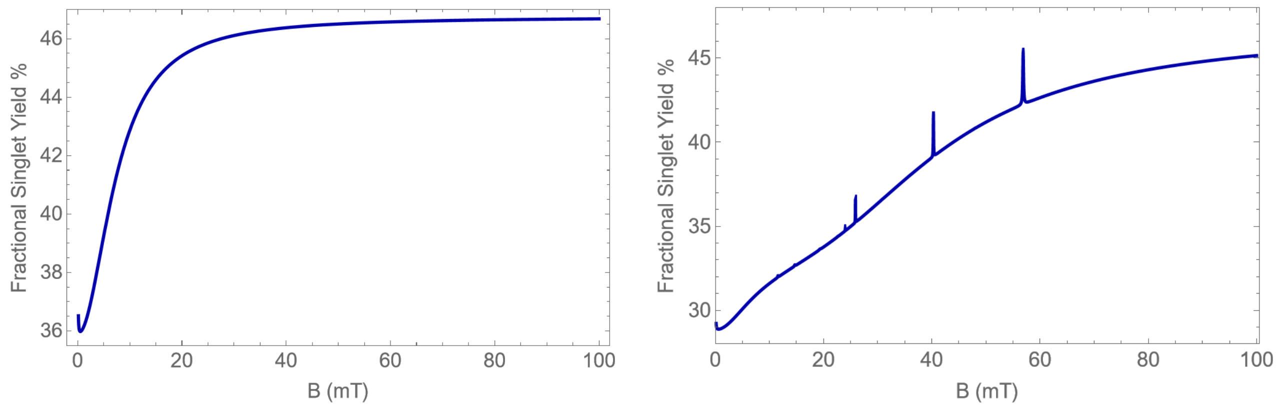

(a) (b)

Figure 2. Variation of the fractional singlet yield percentage with magnetic field strength ranging from 0.15 mT to 100 mT for the Tyr Pathway. The figure is plotted for the choice of $k = 2 \times 10^6$ s^{-1} and $r = 3 \times 10^5$ s^{-1}: (a) System containing only ^{24}Mg and ^{26}Mg isotopes is considered; (b) System containing ^{25}Mg isotope with the HFCC, $a_B = -11.22$ mT has been considered.

Tyr Pathway

Similar plots of the quantity S as in the previous section were plotted for the radical pair involving the tyrosine oxyradical in Fig. 3. We consider the HFCC contribution from the hydrogen nucleus of the Tyrosine radical having the highest HFCC, $a_A = 1.86$ mT, for all the plots.

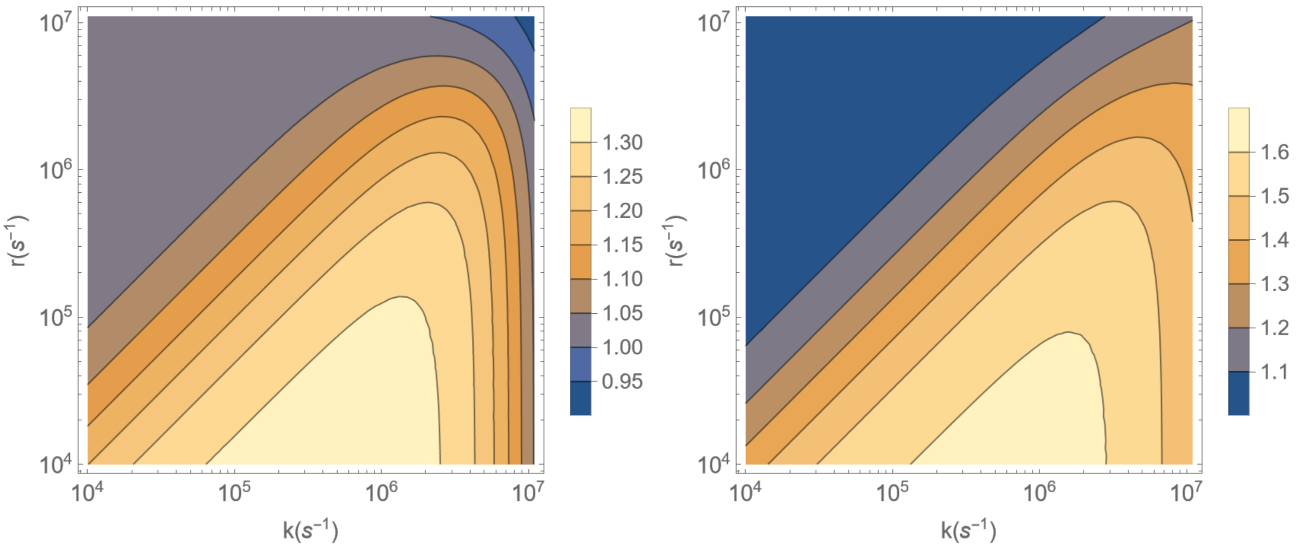

(a) (b)

Figure 3. Ratio of the fractional singlet yield percentages, S plotted against $k(\mathrm{s}^{-1})$ and $r(\mathrm{s}^{-1})$ for Tyr radical case: (a) System containing only ^{24}Mg and ^{26}Mg isotopes is considered; (b) System containing ^{25}Mg isotope with the HFCC, $a_B = -11.22$ mT has been considered.

Figs. 4a and 4b show examples of the variation of fractional singlet yield percentage with magnetic field strength from 0.15 mT to 100 mT for the choice of $k = 2 \times 10^6$ s^{-1} and $r = 2 \times 10^5$ s^{-1} which fall in the range of possible values of k and r.

The differences that one observes between the spinful and spinless isotopes for this case are similar to those for the Ser pathway.

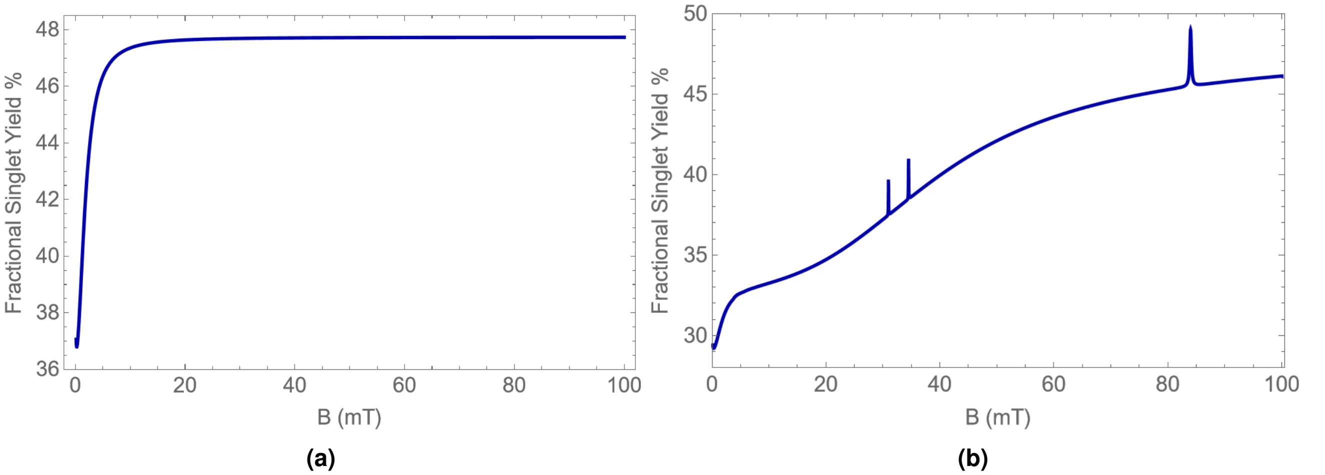

Figure 4. Variation of the fractional singlet yield percentage with magnetic field strength ranging from 0.15 mT to 100 mT for the Tyr Pathway. The figure is plotted for the choice of $k = 2 \times 10^6$ s^{-1} and $r = 2 \times 10^5$ s^{-1}: (a) System containing only ^{24}Mg and ^{26}Mg isotopes is considered; (b) System containing ^{25}Mg isotope with the HFCC, $a_B = -11.22$ mT has been considered.

B. Isotope Contributions at Low Field

We plot examples in Fig.5 from both the Ser and Tyr pathway by considering the contribution from the spinless and spinful isotopes separately to understand the contribution of the same to the drop in FSY at MF strength close to 0 mT. We find that the drop in FSY values occur with or without the contribution from the ^{25}Mg isotope.

C. Considering $T_1 \neq T_2$

For radical pairs in a non-zero magnetic field, the longitudinal and transverse paramagnetic relaxation times, T_1 and T_2, tend to be different. In this situation, the singlet state population is given by

$$\rho_{ss}(t) = \frac{1}{4} + \frac{1}{4}e^{-t/T_1} + e^{-t/T_2}\left(\rho_{ss}^0(t) - \frac{1}{2}\right),$$

where $\rho_{ss}^0(t)$ is the singlet state population calculated ignoring paramagnetic relaxation[1].
Considering this equation to calculate the singlet yield, Φ_S', we get:

$$\Phi_S' = \frac{1}{4} + \frac{k}{4(r_1+k)} - \frac{k}{2(r_2+k)} + \frac{1}{M}\sum_{m=1}^{4M}\sum_{n=1}^{4M} |\langle m|\hat{P}^S|n\rangle|^2 \frac{k(k+r_2)}{(k+r_2)^2+(\omega_m-\omega_n)^2}.$$

It is usually found that $T_1 \geq T_2$ for most systems. Many papers studying the relaxation properties of organic radicals show that this is typically the case, and also show that the relaxation times tend to be around 0.1 - few 10s μs[2–5] as measured at approximately 300mT, which fits the range of relaxation rates we consider.

Based on this, we illustrate the behaviour of our system based on the new Φ_S' equation with an example choice of r_1 in Figs. 6 & 7 for the Tyr Pathway. These results also show a similar behaviour to what we have predicted in the paper.

D. HFCCs of Other Nuclei

For the calculations in this paper, we consider only the HFI contribution of the nuclei of the oxyradical with the highest HFCC. This is a common approximation that is used in such calculations for the sake of simplicity.

The next highest HFCC of a nucleus in the Serine oxyradical was 5.73 mT. Similarly, in the case of the Tyrosine oxyradical, the next highest HFCCs were -0.64 mT and -0.53 mT.

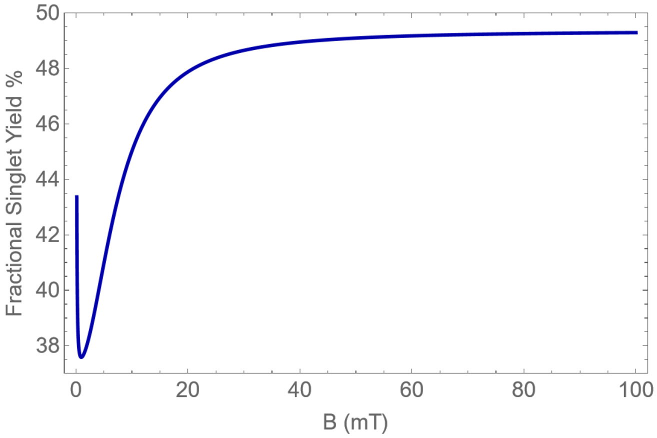

(a) Ser Pathway

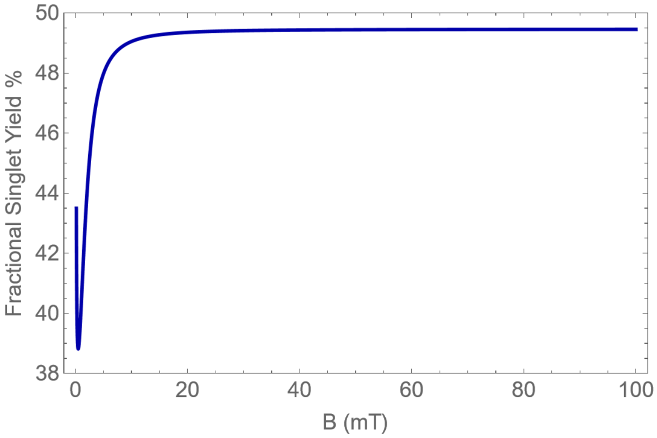

(b) Tyr Pathway

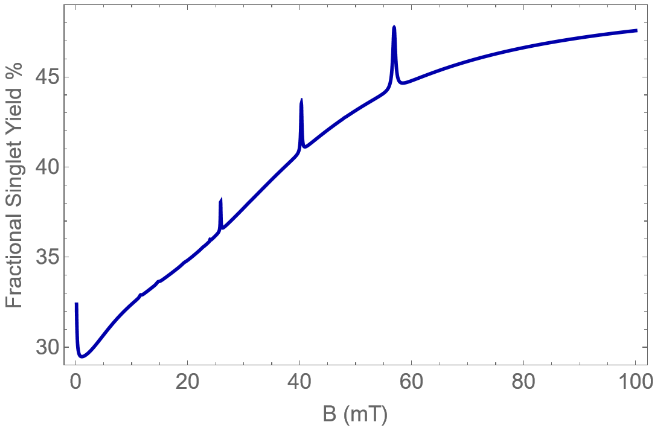

(c) Ser Pathway

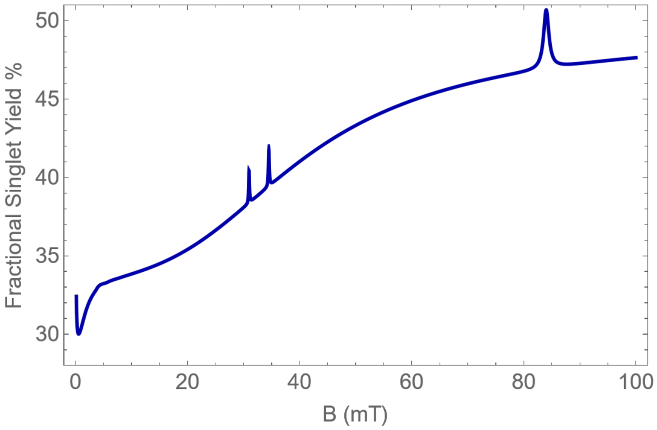

(d) Tyr Pathway

Figure 5. Variation of the FSY percentage with magnetic field strength ranging from 0.15 mT to 100 mT plotted with $k = 7.5 \times 10^6$ s^{-1} and $r = 2 \times 10^5$ s^{-1}. (a) and (b) are plotted considering a system with only ^{24}Mg and ^{26}Mg isotopes; (c) and (d) are plotted considering system with only ^{25}Mg isotope at its natural abundance of 10%. The HFI contribution from the same is $a_B = -11.22$ mT.

References

1. Bagryansky, V. A., Borovkov, V. I. & Molin, Y. N. Quantum beats in radical pairs. *Russ. Chem. Rev.* **76**, 493–506, DOI: 10.1070/rc2007v076n06abeh003715 (2007).
2. Eaton, S. S. & Eaton, G. R. *Relaxation Times of Organic Radicals and Transition Metal Ions*, 29–154 (Springer US, 2002).
3. Tadyszak, K., Mrówczyński, R. & Carmieli, R. Electron spin relaxation studies of polydopamine radicals. *The J. Phys. Chem. B* **125**, 841–849, DOI: 10.1021/acs.jpcb.0c10485 (2021).
4. Yong, L. *et al.* Electron spin relaxation of triarylmethyl radicals in fluid solution. *J. Magn. Reson.* **152**, 156–161, DOI: 10.1006/jmre.2001.2379 (2001).
5. Meyer, V., Eaton, S. S. & Eaton, G. R. Temperature dependence of electron spin relaxation of 2, 2-diphenyl-1-picrylhydrazyl in polystyrene. *Appl. Magn. Reson.* **44**, 509–517, DOI: 10.1007/s00723-012-0417-7 (2012).

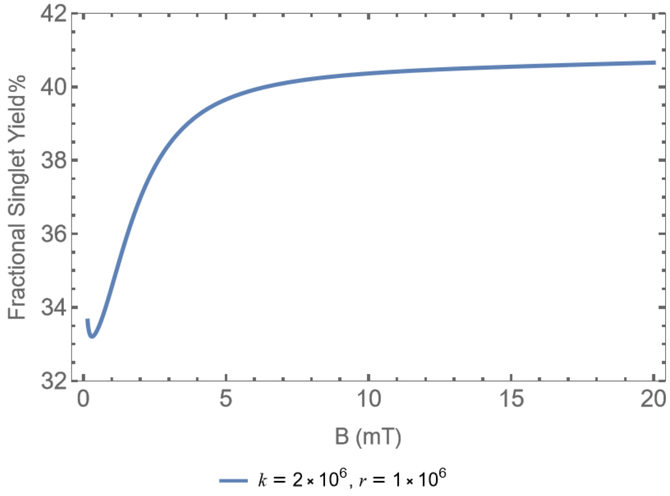

Figure 6. Fractional Singlet Yield % plotted at low magnetic field strength for the Tyr Pathway, considering the original Φ_S equation from the paper.

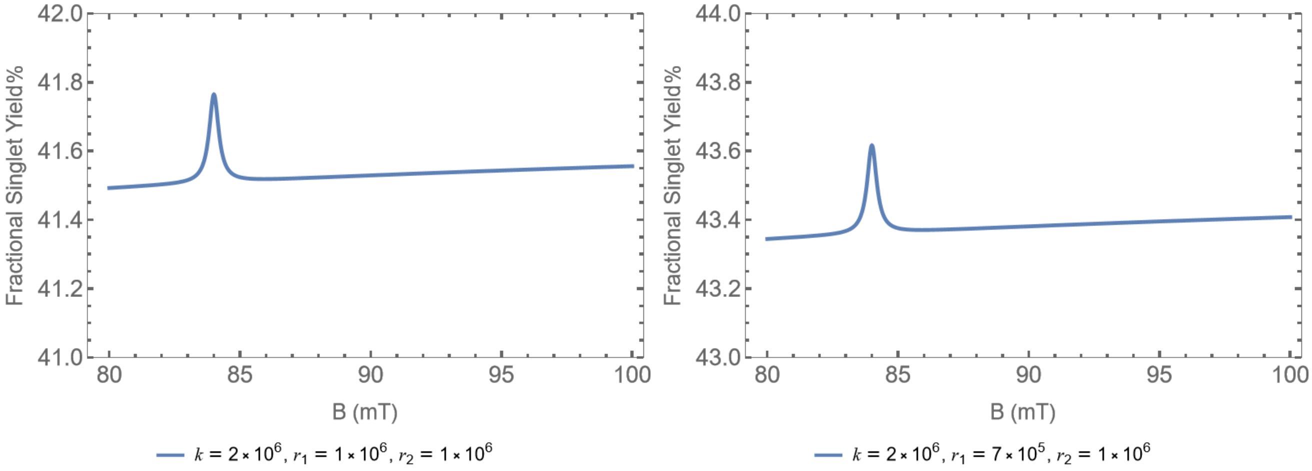

S = 1.24 S = 1.29

Figure 7. Fractional Singlet Yield % plotted considering the Φ'_S from $B = 80$ mT to 100 mT.
